# Serial assessments of cardiac output and mixed venous oxygen saturation in comatose patients after out-of-hospital cardiac arrest

**DOI:** 10.1186/s13054-023-04734-w

**Published:** 2023-11-20

**Authors:** Tobias Zimmermann, Pedro Lopez-Ayala, Mervyn Singer

**Affiliations:** 1 Bloomsbury Institute of Intensive Care Medicine, University College London, London, UK; 2 Cardiovascular Research Institute Basel, University Hospital Basel, University of Basel, Basel, Switzerland

We read with great interest the article “Serial assessments of cardiac output and mixed venous oxygen saturation in comatose patients after out-of-hospital cardiac arrest” by Grand et al. [1] While the reported findings are generally in line with what we would predict from a physiological point of view, we query the rigor of the statistical analyses.

To illustrate the relationship between mortality and hemodynamic variables, the authors used a Cox proportional hazards model with smoothing splines to attain the predicted values. It is unclear whether this was an univariable or multivariable model. The authors then present Figure 4 of their paper as a plot of the “Hazard ratio of mortality as a function of first measured mixed venous oxygen saturation […] and first measured cardiac index […] values during intensive care after cardiac arrest.” However, since no reference point was chosen, this represents a (relative) hazard plot and not a hazard ratio plot [2]. A dose–response plot with an appropriate reference point (illustrated in Fig. 1), defined based on prior clinical knowledge, should be presented instead. The literature offers examples of such hazard ratio dose–response plots [3]. This could potentially affect the conclusion that can be drawn; for example, depending on the location of the chosen reference point, a significant association between cardiac index and mortality might be revealed.

**Fig. 1:**
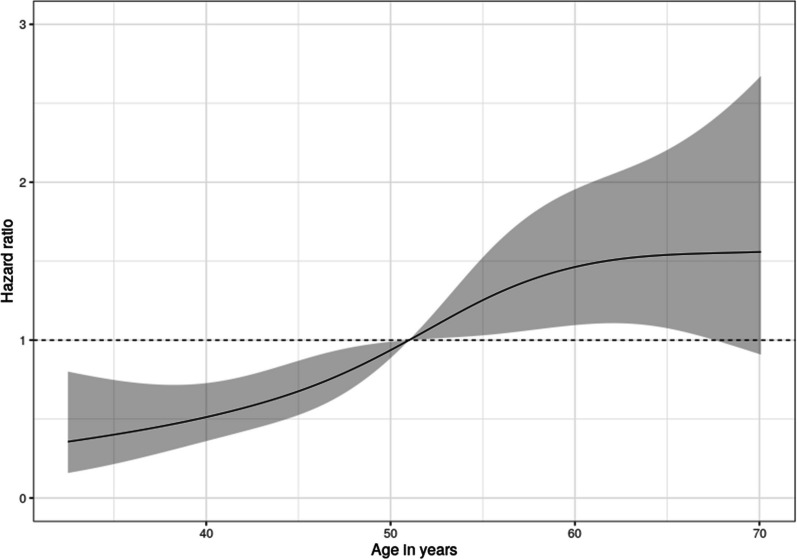
Example of a dose–response hazard ratio plot based on the open access Mayo Clinic Primary Biliary Cholangitis Dataset. A multivariable Cox proportional hazards model was fitted, conditioning on treatment received and bilirubin level. To relax the linearity assumption, restricted cubic splines with four knots were fitted for age and bilirubin. Note the selected reference point where the 95% CI collapses. The R code for creating this figure can be found online (https://gitlab.com/tobiaszimmermann/ltte_critcare_grand_2023)

Could the authors also elaborate on their choice of relaxing linearity assumptions with splines for graphical illustration but not for the regression models? Seeing the nonlinear relationship between the variables of interest and the outcome graphically represented in Figure 4 of their paper, should this not be taken into account when fitting the multivariable prediction model? Categorizing cardiac index into quarters is a suboptimal way of dealing with nonlinearity. This should be avoided [4] especially as splines have already been introduced into the analysis that are capable of relaxing linearity assumptions within prediction models [2, 5]. This could have important implications on the central conclusions drawn from the analysis.

## Data Availability

The Mayo Clinic Primary Biliary Cholangitis Dataset used to create the example of a dose–response hazard ratio plot is part of the R “survival” package (https://rdrr.io/cran/survival/man/pbc.html) [6]. The R code for creating this figure can be found online (https://gitlab.com/tobiaszimmermann/ltte_critcare_grand_2023).
